# Combining promoter and induced intron 1 mutations of *Vrn-A1a* does not accelerate flowering in wheat

**DOI:** 10.1007/s11032-026-01657-6

**Published:** 2026-04-09

**Authors:** Beáta Strejčková, Zbyněk Milec, Tereza Šlajsová, Vojtěch Hudzieczek, Rocío Alarcón-Reverte, Caroline A. Sparks, Ales Pecinka, Stephen Pearce, Jan Šafář

**Affiliations:** 1 Institute of Experimental Botany, Czech Acad Sci, Centre of Plant Structural and Functional Genomics, Olomouc, 779 00 Czech Republic; 2 Global Change Research Institute of the Czech Academy of Sciences, Bělidla 986/4a, Brno, 603 00 Czech Republic; 3 Department of Plant Developmental Genetics, Institute of Biophysics, Czech Acad Sci, Brno, 612 65 Czech Republic; 4 Rothamsted Research, Harpenden, Hertfordshire AL5 2JQ UK

**Keywords:** Wheat, Vernalization, *Vrn-A1a*, RIP3, Genome editing, Crops

## Abstract

In temperate cereals, vernalization regulates the transition from vegetative to reproductive growth in response to low temperatures. A central component of this pathway is *VERNALIZATION 1* (*VRN1*). In winter wheat cultivars, the recessive *vrn1* allele is transcriptionally activated by cold and promotes the transition to flowering. In contrast, spring cultivars carry dominant *VRN1* alleles expressed in a cold-independent manner. This is caused by mutations in the *cis*-regulatory elements in the promoter or in the “critical region” of intron 1. The widespread spring bread wheat allele *Vrn-A1a* carries a promoter mutation but retains a winter-like allele intron 1. Intriguingly, *Vrn-A1a* is partially responsive to cold, suggesting regulatory element(s) beyond the promoter mutation. Here, we investigated the effect of combined promoter and first intron mutations on *Vrn-A1a* expression and flowering time in bread wheat. We deleted a 901-bp segment of the *Vrn-A1a* so-called “critical region”, thus generating a novel combination of the promoter and intron 1 mutations not found in natural germplasm. Plants carrying both mutations exhibited *Vrn-A1* transcript levels and flowering times comparable to those of the wild-type under both vernalizing and non-vernalizing conditions. Hence, partial cold responsiveness of *Vrn-A1a* is controlled by regulatory element(s) outside the removed intron 1 segment. Overall, our study advances the understanding of *VRN1* transcriptional regulation and highlights the complexity of vernalization control in wheat.

**Supplementary Information:**

The online version contains supplementary material available at 10.1007/s11032-026-01657-6.

In temperate cereals such as bread wheat (*Triticum aestivum* L.), the transition from vegetative to reproductive growth in winter varieties is controlled by the vernalization pathway, with the MADS-box transcription factor VRN1 serving as the central player. Recessive *vrn1* alleles in winter cultivars are activated upon prolonged cold exposure (Yan et al. 2003, 2004; Fu et al. 2005). In contrast, spring *VRN1* alleles typically carry dominant mutations in the promoter or the first intron that allow cold-independent activation, conferring early flowering even in allopolyploid bread wheat. Regulation of *VRN1* involves a multilayered network integrating transcriptional, post-transcriptional, and sequence-variation-based mechanisms (reviewed in (Milec et al. 2023).

*VRN1* is transcriptionally regulated through chromatin modifications and likely also by a higher-order 3D chromatin organization (Liu et al. 2024b). In winter cultivars, which likely retain the complete set of *VRN1 cis*-regulatory regions, vernalization leads to a decrease in the repressive modification histone H3 lysine 27 tri-methylation (H3K27me3) and an increase in the activating modification histone H3 lysine 36 tri-methylation (H3K36me3) at the *VRN1* promoter and first intron (Liu et al. 2024a). Furthermore, the winter recessive alleles are proposed to be repressed by a chromatin loop between specific motifs in the promoter and the first intron before vernalization, which gradually relaxes during cold exposure (Xu et al. 2021). During the early vernalization period, a non-coding sense *VRN1* alternative splicing (VAS) transcript is produced exclusively in winter cultivars. VAS enhances *VRN1* expression by recruiting the RF2a/RF2b transcription-factor heterodimer to the promoter. With progressing vernalization, the loop relaxation coincides with an increased *VRN1* mRNA accumulation (Xu et al. 2021).

Besides promoter mutations, a conserved wheat-barley ∼2.8 kb segment in the first intron plays a central role in *VRN1* sequence-based regulation (von Zitzewitz et al. 2005; Szucs et al. 2007). Deletions within this region are tightly associated with spring growth habit across temperate cereals (Fu et al. 2005; von Zitzewitz et al. 2005). In recessive alleles, this region is intact and participates in vernalization-dependent activation. This “critical region” contains multiple conserved motifs, including light and cold-responsive motifs, Dof protein binding sites, RNA Immune Precipitation fragment 3 (RIP3), and G-box-like sequences (von Zitzewitz et al. 2005). A working model proposes that, before vernalization, the wheat GLYCINE RICH RNA-BINDING PROTEIN 2 (GRP2) associates with the RIP3 motif in the first intron of *VRN1* pre-mRNA to limit transcript accumulation (Kippes et al. 2015). Natural polymorphisms at RIP3 correlate with differences in vernalization requirement and heading time among winter wheat lines, consistent with the proposed regulatory role (Kippes et al. 2015, 2018). In addition, an early cold-responsive *VRN-A1* short splice variant, initiating at the canonical transcription start site and joining exon 1 to a short intron 1 segment that includes RIP3, has been reported (Kippes et al. 2018). These results suggest that the RIP3-containing segment of intron 1 plays a significant role in *VRN1* regulation. While these studies focused on winter backgrounds, the structure and regulation of the dominant spring allele *Vrn-A1a* provide an intriguing contrast that may illuminate how promoter and intron elements interact.

The spring *Vrn-A1a* allele retains the entire first-intron critical region, yet still drives strong constitutive expression. *Vrn-A1a* is the most frequent spring allele in hexaploid wheat, and can occur in one or two copies (Yan et al. 2004; Strejčková et al. 2021). It carries a duplication that spans part of the proximal promoter and exon 1 (including the region around the “spring foldback element” (SFE) insertion), extending into the first portion of intron 1 (Yan et al. 2004). Lines with *Vrn-A1a* show high basal *VRN1* transcript levels and therefore do not require vernalization (Pugsley 1971; Loukoianov 2005). However, cold treatment can further increase *Vrn-A1a* expression (Alonso-Peral et al. 2011; Li et al. 2024), suggesting residual vernalization responsiveness mediated by as yet-unidentified *cis*-regulatory element(s), possibly within intron 1.

Within this framework, we tested whether removing the RIP3-containing segment of intron 1 critical region in a *Vrn-A1a* spring background leads to further derepression of its expression, reduces residual vernalization sensitivity, and promotes earlier flowering.

We generated a *Vrn-A1a*^*Δ901*^ line in spring bread wheat Cadenza using CRISPR/Cas9 to delete a 901-bp segment from the first intron of *Vrn-A1a* encompassing the RIP3 region, a putative TaGRP2 repressor-binding site (Fig. 1a, Fig. S1). Plants were grown in growth chambers under long-day (LD, 21 °C 14 h day/18°C 10 h night) conditions. To test whether the loss of this element affects flowering, sibling *Vrn-A1a* and *Vrn-A1a*^*Δ901*^ lines were grown under non-vernalizing LD (V0) and after 14 (V14) or 28 (V28) days of vernalization in the growth chamber (6 °C 8 h day/6°C 16 h night; Fig. 1b). All seeds were germinated at the same time and grown in parallel, with both vernalization treatments starting at the same time point (Fig. 1b). No visible phenotypic differences were observed between the edited and wild-type plants after the given treatment. Apex dissection at 45 days after germination revealed no developmental differences in the inflorescence (Fig. 1c), and heading time was not significantly different between *Vrn-A1a* and *Vrn-A1a*^*Δ901*^ genotypes in any treatment (Fig. 1d).

**Fig. 1:**
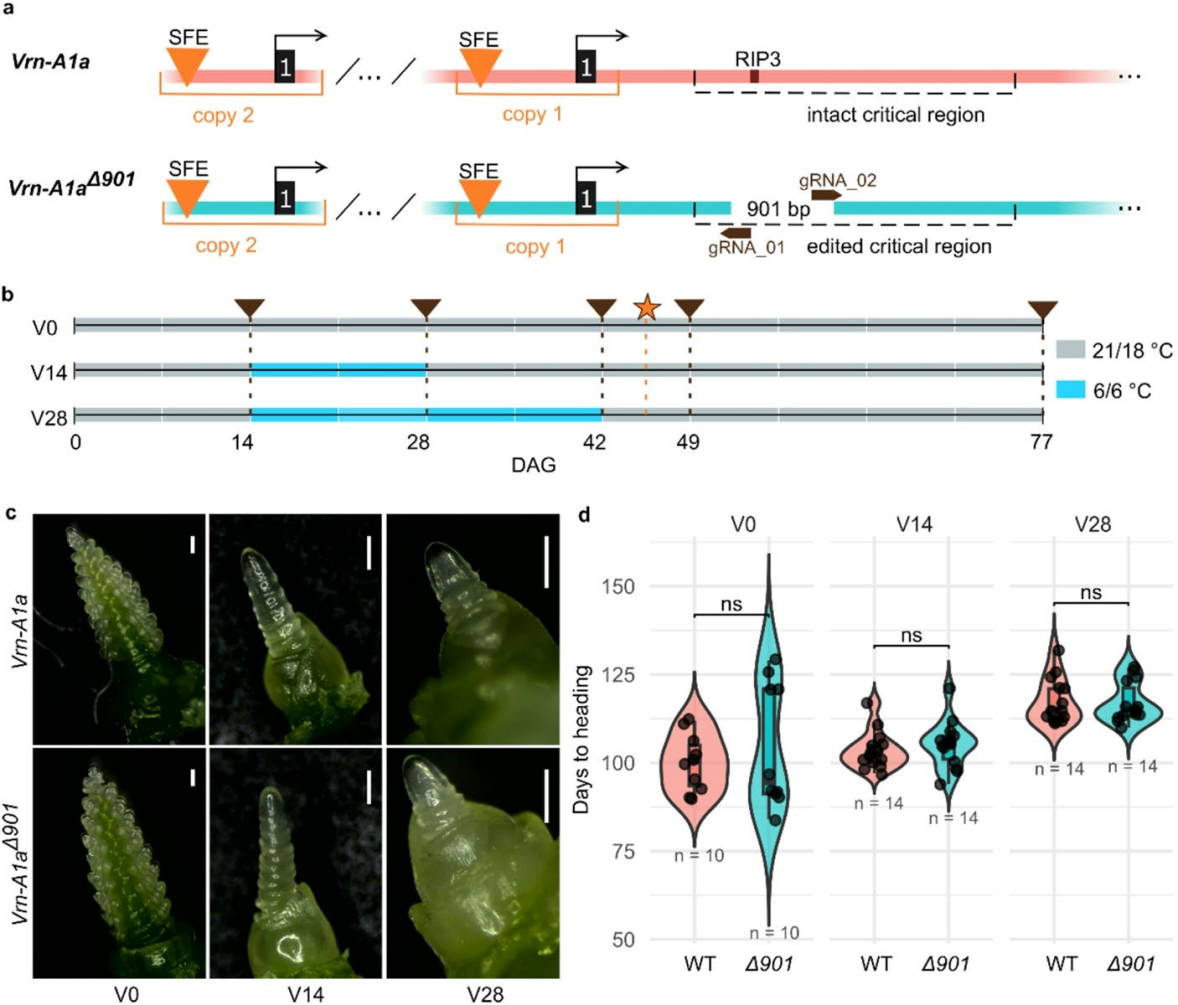
The*Vrn-A1a*^*Δ901*^allele does not affect flowering time under various vernalization regimes.** a** Schematic representation of the promoter and first intron regions of the wild-type *Vrn-A1a* and CRISPR/Cas9-edited *Vrn-A1a*^*Δ901*^ allele with the 901-bp deletion located within the “critical region”. SFE – spring foldback element, black rectangles – exons, RIP3 – RNA Immune Precipitation fragment 3. The scheme is not to scale. **b** The three different growing conditions and sampling timepoints of *Vrn-A1a* and *Vrn-A1a*^*Δ901*^ plants. V0/V14/V28 indicate vernalization treatments for 0, 14 and 28 days, respectively, with day/night temperatures. Brown triangles – leaf sampling for RNA isolation, orange star – shoot apical meristems (SAMs) dissection, DAG – days after germination. **c** Developmental stages of SAMs of *Vrn-A1a* and *Vrn-A1a*^*Δ901*^ plants under V0, V14, and V28 conditions at 45 DAG. **d** Heading time of the *Vrn-A1a*^*Δ901*^ (*Δ901*) and wild-type (WT) *Vrn-A1a* plants under V0, V14, and V28 conditions. Statistics: T-test, FDR-corrected. ns = not significant at *P*^*adj*^*< 0.05*

Analysis of *Vrn-A1a* expression at five time points (Figs. 1b and 2) confirmed significant induction by vernalization (two-way ANOVA, F(2, 84) = 3.57, *P* = 0.033), with higher levels under V28 compared with V0 (Tukey’s post-hoc test, *P* = 0.036). There was no significant effect of genotype (F(1, 84) = 2.83, *P* = 0.096) and no genotype × treatment interaction (F(2, 84) = 0.17, *P* = 0.84), indicating similar responses in both genotypes. Pairwise t-tests at each time point within each treatment, corrected by false discovery rate (FDR), also revealed no differences between the *Vrn-A1a* and *Vrn-A1a*^*Δ901*^ line (all FDR-adjusted *P* > 0.05; Fig. 2). Thus, the induced deletion did not alter *Vrn-A1a* expression relative to wild-type under any of the tested growth conditions.

**Fig. 2:**
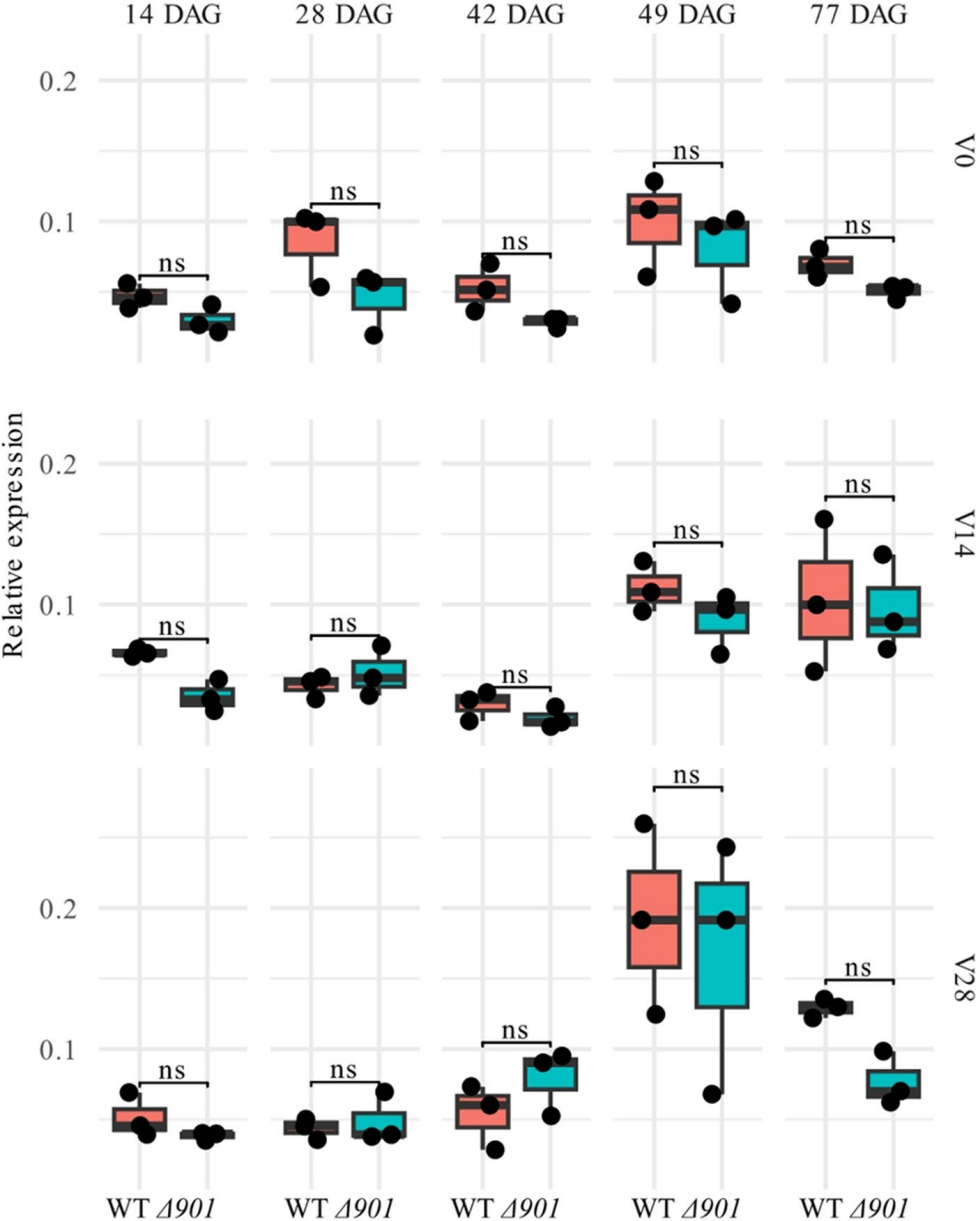
The *Vrn-A1a*^*Δ901*^ allele does not alter *Vrn-A1* mRNA levels. Relative *Vrn-A1* mRNA levels in leaves of *Vrn-A1a*^*Δ901*^ (*Δ901*) and wild-type (WT) *Vrn-A1a* lines at five different days after germination (DAG) and in three treatments (V0, V14, V28). Individual points represent three biological replicates. Statistics: T-test, FDR-corrected. ns = not significant at *P*^*adj*^ *< 0.05*

Our results suggest that simultaneous interference with both the promoter and intron 1 regions does not further activate *Vrn-A1a* in this genetic background under the applied experimental conditions. The dominant *Vrn-A1a* allele carries a promoter–exon 1 duplication and an SFE promoter insertion. Speculatively, this structural variation likely shifts *VRN-A1* regulation toward promoter-driven transcription. Under this model, the promoter-level control conferred by the duplication could buffer the loss of the RIP3 site-containing segment. Moreover, the RIP3/intron 1 mutations might be epistatic to the promoter mutations, as these regulatory mechanisms are not independent but act within the same functional complex. A vernalization-sensitive chromatin loop connecting the promoter and intron 1 motifs has been reported (Xu et al. 2021), supporting the idea that both regions cooperate in *VRN1* transcriptional regulation, and that mutation of either region could disrupt this mechanism.

Our observation that *Vrn-A1a* transcript level increases after prolonged cold exposure is consistent with earlier studies (Alonso-Peral et al. 2011; Li et al. 2024). In the spring wheat Fielder vernalized for 28 days, ChIP-qPCR revealed increased H3K4me3 and decreased H3K27me3 at two intron 1 regions (Li et al. 2024). Alignment of the regions defined by the qPCR primer sequences to Cadenza *Vrn-A1a* showed that they are located 1,427 bp upstream and 291 bp downstream of the deleted segment in *Vrn-A1a*^*Δ901*^. Although ChIP-qPCR lacks genome-wide or high-resolution coverage, these results suggest histone modification changes across a broader regulatory region, including the RIP3 site. However, the *Vrn-A1a*^*Δ901*^ allele remained responsive to vernalization even after deleting the 901-bp region in the first intron (Fig. 1a). This suggests that elements outside the deleted sequence – such as promoter or other intronic regions – are sufficient to mediate cold responsiveness of *Vrn-A1a*.

It has been proposed that the GRP2 repressor limits *VRN1* mRNA accumulation in winter wheat prior to cold by binding to the RIP3 region in *VRN1* intron 1. We hypothesized that deleting the RIP3-containing segment could further elevate *Vrn-A1a* expression levels and hasten flowering. We demonstrated that a 901-bp deletion, created in the *Vrn-A1a* first intron and removal of the RIP3-containing region, had no detectable effect on *Vrn-A1a* ^*Δ901*^ transcript abundance or heading time under either non-vernalizing or vernalizing regimes in the spring wheat Cadenza background. These results highlight the context-dependent role of RIP3. It appears to be critical for vernalization responsiveness in winter wheat, but dispensable in spring *Vrn-A1a*, where the promoter mutation acts as a significant effect *cis*-regulatory element.

We conclude that our results align with the two distinct models for *VRN1* activation: a promoter/transcription start site (TSS) model and a first-intron model. In *Vrn-A1a*, the promoter model dominates, while in *Vrn-B1/D1*, deletion of intron 1 repression is sufficient to confer a spring growth habit. In other words, spring growth habit can arise via either promoter mutations (*Vrn-A1a*) or large intron 1 deletions that remove putative *cis*-regulatory elements (e.g., *Vrn-A1c/B1/D1*); however, combining both mutations is non-additive.

## Supplementary Information

Below is the link to the electronic supplementary material.

Supplementary file 1. Figure S1,Table S1 and Methodology.

## Data Availability

The datasets generated and analysed during the current study are available from the corresponding author upon request.
