# Supplementary material for: Combining promoter and induced intron 1 mutations of Vrn-A1a does not accelerate flowering in wheat
Source: Mol Breed. 2026 Apr 9;46(4):33. doi: 10.1007/s11032-026-01657-6 (PMC13065874; doi:10.1007/s11032-026-01657-6)
Supplement: Supplementary file 1 — Figure S1,Table S1 and Methodology. [file 11032_2026_1657_MOESM1_ESM.docx]

**Supplementary file 1 for:**

**Combining promoter and induced intron 1 mutations of *Vrn-A1a* does not accelerate flowering in wheat**

Beáta Strejčková^1^ (ORCID: 0000-0003-3093-0957), Zbyněk Milec^1,2^ (ORCID: 0000-0003-4724-9519), Tereza Šlajsová^1^, Vojtěch Hudzieczek^3^ (ORCID: 0000-0002-7468-7197), Rocío Alarcón-Reverte^4^ (ORCID: 0009-0009-5962-3943), Caroline A. Sparks^4^, Ales Pecinka^1^ (ORCID: 0000-0001-9277-1766), Stephen Pearce^4^ (ORCID: 0000-0002-1794-7618), Jan Šafář^1,^* (ORCID: 0000-0003-0197-1979)

^1^Institute of Experimental Botany, Czech Acad Sci, Centre of Plant Structural and Functional Genomics, 779 00 Olomouc, Czech Republic. ^2^Global Change Research Institute of the Czech Academy of Sciences, Bělidla 986/4a, 603 00 Brno, Czech Republic, ^3^Institute of Biophysics, Czech Acad Sci, Department of Plant Developmental Genetics, 612 65 Brno, Czech Republic. ^4^Rothamsted Research, Harpenden, Hertfordshire, AL5 2JQ, UK.

*Corresponding author: safar@ueb.cas.cz

**Contents**

Table S1

Figure S1

Methodology

**Table S1. gRNA and PCR primer sequences used in this study.**

| Purpose | Target | | Sequence 5 'to 3 ' | Reference |
| --- | --- | --- | --- | --- |
| gRNAs for Cas9 | | | |  |
| gRNA_01 | | *Vrn-A1a* | CTGAGGTGTGATTCCATCAG | This study |
| gRNA_02 | |  | GAGTCAAGGTACTAAAAAAG |  |
| Genotyping primers | | | |  |
| VRN-A1a_F1 | *Vrn-A1a* | | AACCAGTTATCCTCTACACCTATTG | This study |
| VRN-A1a_R1 |  |  | TCTAGGCTCCGCACCATTGATT |  |
| VRN-A1a_F2 |  |  | CTGGAACGTCAGGAATGTCG |  |
| VRN-B1_F1 | *vrn-B1* | | CATGCATGTGTTGTCGGTCTAT | This study |
| VRN-B1_R1 |  |  | CACCACTGATACCTCCCTCCG |  |
| VRN-D1_F1 | *vrn-D1* | | CACCTATATATTGTGTCCCTAAACCTAG | This study |
| VRN-D1_R1 |  |  | ACACATCACCATTGTCGCAC |  |
| Cas9_F1 | *Cas9* | | AAGGCTATCGTGGACCTCCT | This study |
| Cas9_R1 |  |  | GTCTTCCCGGACTGCTTGT |  |
| RT-qPCR primers | | | |  |
| q.VRNA1-L-F1 | *VRN-A1* full transcript | | TCCACCGAGTCATGTATGGA | Kippes *et al*., 2018[10] |
| q.VRNA1-L-R1 |  |  | GAGAACCTTTTCTGCATAAGAA |  |
| GAPDH_F | *GAPDH* | | TTAGACTTGCGAAGCCAGCA | Sun *et al*., 2014[20] |
| GAPDH_R |  |  | AAATGCCCTTGAGGTTTCCC |  |

**Fig. S1****. Pairwise alignment of the first intron fragment from the wild-type Vrn-A1a (1) and CRISPR/Cas9-edited *Vrn-A1a^Δ901^* (2) allele in cultivar Cadenza.** The alignment shows two single nucleotide polymorphisms and a 901-bp deletion (teal) in the mutant allele. Identical bases are shaded in black. RIP3 – RNA Immune Precipitation fragment 3, PAM - protospacer adjacent motif.


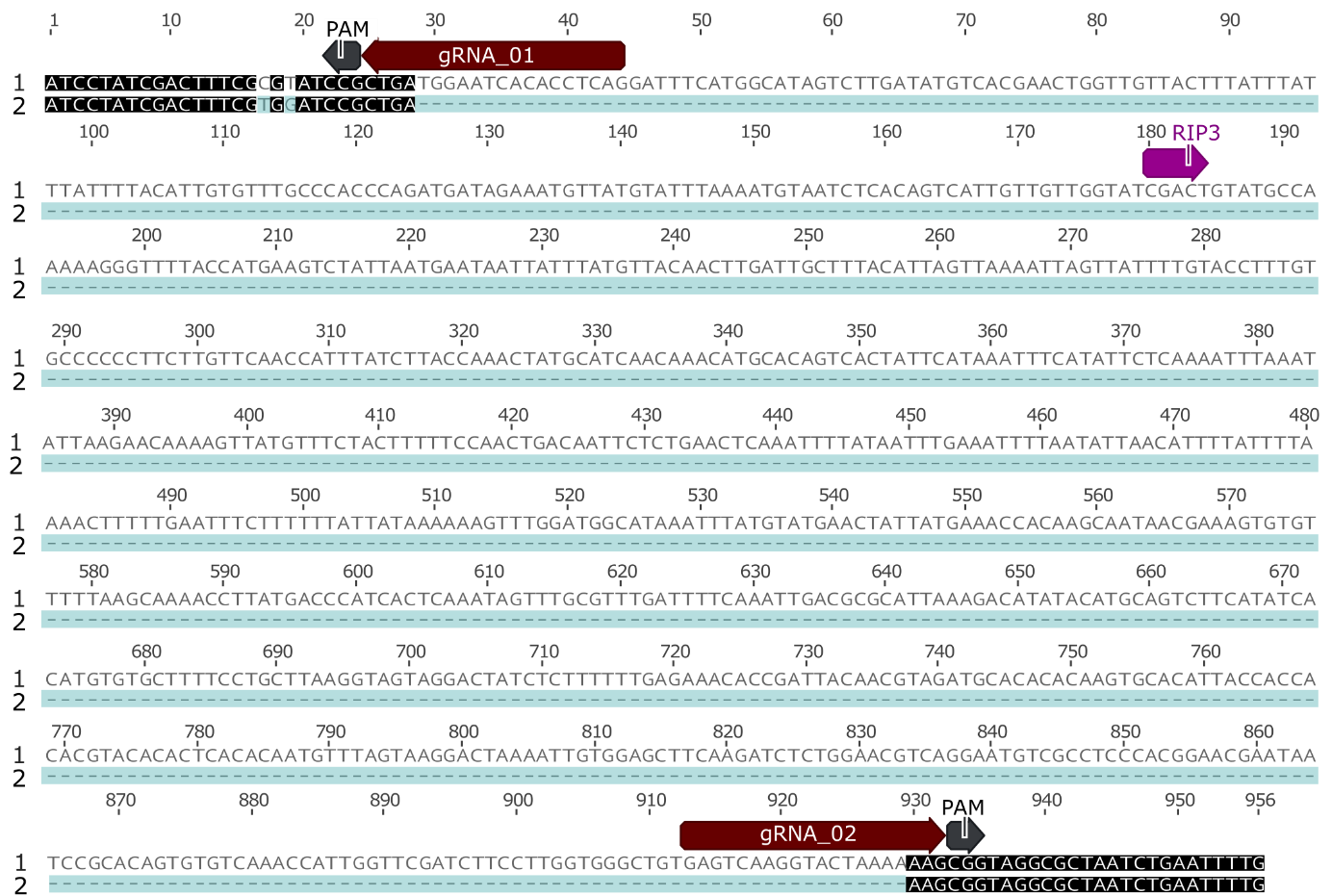


**Methodology**

- 1. Plant materials and growth conditions

All experiments were performed in the spring bread wheat (*Triticum aestivum* L.) cultivar Cadenza. Before planting, the seeds were stratified for three days at 4 °C and germinated on Petri dishes with wet filter paper at 24 °C in the dark. Plants were grown in growth chambers under long-day (LD, 21°C 14 h day/18 °C 10 h night) conditions. Vernalization was conducted in the growth chamber (6°C 8 h day/6 °C 16 h night) for either 14 (V14) or 28 (V28) days.

For the experiments, 15 replicates of mutant (CRISPR/Cas9-edited *Vrn-A1a^Δ901^* allele) and a sibling line lacking the T-DNA insertion (wild-type *Vrn-A1a* allele) were grown under three different growth conditions: V0, V14, and V28. V0 plants remained in non-vernalizing LD conditions for the whole experiment, whereas 14-day-old plants were vernalized for 14 and 28 days, respectively, and then returned to LD. Leaves for RNA isolation were sampled from V0, V14, and V28 plants at five time points: 14, 28, 42, 49, and 77 days after germination (DAG). At each time point, the youngest fully developed leaf that was present in all sampled plants was collected to ensure developmental consistency across samples. Shoot apical meristems from V0, V14, and V28 plants were dissected at 45 DAG and documented via an Olympus SZ61 Stereo Microscope equipped with a camera. Heading time was scored when half of the spike emerged from the flag leaf.

- 1. Generation of *Vrn-A1a^Δ901^* allele

Two sgRNAs flanking the *Vrn-A1a* intron 1 region containing the RIP3 site were designed to induce a deletion of around 1 kb (Table S1). sgRNAs complementary to *VRN-B1* and *VRN-D1* homoeologous sequences were excluded. Instead, gRNA_01 with two SNPs and gRNA_02 with four SNPs relative to both *VRN-B1* and *VRN-D1* were used. Cloning was performed as described in (Čermák et al. 2017). Briefly, two sgRNA spacers targeting the desired sequence were cloned downstream of two *TaU6* promoters in module B (*pMOD_B2518*) and C (*pMOD_C2518*) vectors. Next, the intermediate module plasmids A (*pMOD_A1110* carrying *ProZmUbi1:TaCas9*), B, and C were assembled into the transformation backbone *pTRANS_160* containing *ProPvUbi2:bar* selection marker. Genetic transformation of Cadenza was performed at the Crop Transformation and Genome Editing Unit, Rothamsted Research, using a PDS1000/HeTM Biolistic Particle Delivery System (BioRad, Watford, UK), as described (Sparks and Doherty 2020). We generated a *Vrn-A1a^Δ901^* line with a 901 bp deletion in the first intron of *Vrn-A1a* (Figure S1).

As a wild-type control for subsequent experiments, we selected a sibling line emerging from the transformation event that carried the wild-type *Vrn-A1a* allele while segregating out the T-DNA insertion carrying the *Cas9* cassette and gRNA sequences.

- 1. Genotyping and molecular characterization of the transformation progeny

To differentiate between wild-type *Vrn-A1a* and mutant *Vrn-A1a^Δ901^* alleles, we developed a PCR assay that utilizes three primers (VRN-A1a_F1/R1/R2, Table S1), producing amplicons of varying sizes based on the present variant (228 and 1447 bp for *Vrn-A1a*, 546 bp for *Vrn-A1a^Δ901^*). An extension time of 30 s was used to preferentially amplify smaller PCR products. Lines without T-DNA insertions were identified via PCR genotyping using primers targeting the *Cas9* sequence (Table S1).

We confirmed there were no edits in the intron 1 region of *VRN-B1* or *VRN-D1* by Sanger sequencing of homoeologue-specific amplicons from T_0_ plant B3998_R2P3 (Table S1). Primer pair VRN-B1_F1/VRN-B1_R1 amplifies a 1,515 bp amplicon specific to the *VRN-B1*intron 1 region spanning the sgRNA target sites. Primer pair VRN-D1_F1/VRN-D1_R1 amplifies a 1,640 bp amplicon specific to the *VRN-D1*intron 1 region spanning the sgRNA target sites. Both amplicons were amplified by PCR using 65°C annealing temperature and 90 s extension time using genomic DNA from T_0_ plant B3998_R2P3.

The allelic variant (*Vrn-A1a* or *Vrn-A1a^Δ901^*) was confirmed by Sanger sequencing of the amplicons. PCR clean-up was performed using ExoSap (ThermoFisher Scientific, USA). Sequencing reactions were conducted using BigDye Terminator v3.1 Cycle Sequencing Kit (Applied Biosystems, USA) and purified using the Agencourt CleanSEQ Dye-Terminator Removal Kit (Beckman Coulter, USA). The reactions were analyzed on an ABI3730xl DNA analyzer (Applied Biosystems, USA). The resultant sequences were trimmed and assembled using Geneious Prime® software (v2023.0.4; https://www.geneious.com). Pairwise alignment of a first intron fragment from the wild-type *Vrn-A1a* and CRISPR/Cas9-edited *Vrn-A1a^Δ901^* allele was done in Geneious Prime® software (v2023.0.4; https://www.geneious.com) with MAFFT (Katoh and Standley 2013). We screened the T_1_ progeny of the primary transformant for homozygous lines carrying mutations. Homozygous progeny plants (≥ T_2_ generation) were used in the experiments.

- 1. RNA extraction and reverse transcription–quantitative PCR (RT–qPCR)

Total RNA was extracted using the Quick-RNA Miniprep Kit (Zymo Research, USA), with DNase I treatment conducted according to the manufacturer's instructions. cDNA was synthesized using the RevertAid First Strand cDNA Synthesis Kit (Thermo Scientific™, USA), following the manufacturer's instructions, with 1 µg of total RNA and anchored-oligo (dT) 18 primers. Gene expression levels were determined using reverse transcription-qPCR (RT–qPCR). RT–qPCR was performed with qPCR 2x SYBR Master Mix (Top-Bio, Czech Republic) on the CFX96™ Real-Time PCR Detection System (Bio-Rad, USA).

Relative expression values were calculated using the 2^-ΔCq^ method, normalizing Vrn-A1 transcripts to the reference gene GAPDH. Each condition included three biological and two technical replicates. Each biological replicate consisted of a pooled leaf tissue from three individual plants to account for plant-to-plant variability and provide a representative measure of gene expression. Sequences of all primers used for RT–qPCR are listed in Table S1.

- 1. Statistics

Relative expression data (2^-ΔCq^) were tested for normality within each genotype, treatment, and timepoint group using the Shapiro-Wilk test. As the normality assumption was satisfied for all groups (p > 0.05), unpaired two-sample t-tests were performed to compare *VRN-A1* transcript levels in *Δ901* and WT genotypes at each treatment and time point. P-values were adjusted for multiple testing using the Benjamini-Hochberg false discovery rate (FDR) method, with adjusted p-values < 0.05 considered statistically significant. Non-parametric Wilcoxon rank-sum tests were also performed for validation for each comparison. In addition, an overall genotype effect was assessed using two-way ANOVA with genotype, treatment, and their interaction as factors, followed by Tukey's HSD post hoc tests for multiple treatment comparisons.

For each condition, two-tailed t*-*tests were applied to compare heading time between *Δ901* and WT plants. Resulting p-values were corrected for multiple testing using the false discovery rate (FDR) method, and adjusted p-values were annotated for significance.

All statistical analyses were conducted in RStudio (v2021.09.0; https://www.rstudio.com) using the ggpubr and rstatix packages.
